# Markedly Elevated Cardiac Bio-Markers at Presentation With Normal Ventricular Function: A Novel Clinical Subset of Myocarditis Manifestation

**DOI:** 10.4021/cr15w

**Published:** 2011-05-20

**Authors:** Amruth R Palla, Siva Sontineni, Susan Mani

**Affiliations:** a Danbury Hospital, Department of Internal Medicine, USA; b Creighton University School of Medicine, Department of Cardiology, USA; c Danbury Hospital, Department of Cardiology, USA

**Keywords:** Myocarditis, Cardiac troponins, Congestive heart failure, Arrhythmia

## Abstract

We present a case of a 19-year-old woman with myocarditis who had significantly elevated cardiac markers at presentation even before any myocardial damage ensued. The patient had complicated clinical course with ventricular arrhythmia and cardiac arrest requiring resuscitation but eventually recovered completely. Though there is limited information available regarding such cases, the significantly elevated initial cardiac markers in the absence of left ventricular decompensation may probably represent a clinical subset of myocarditis and may portend an impending complicated clinical course. Further systematic research is required to define the clinical phenotype and elucidate underlying mechanisms.

## Introduction

Myocarditis is a commonly diagnosed condition and its patho-physiology is well known. However it is very rare to see a significant elevation of cardiac markers to the extent of troponin I > 100 ng/ml in the early initial phase of the disease especially in the absence of any LV decompensation.

## Case Report

A 19-year-old woman presented with dry cough, shortness of breath, and pleuritic chest pain for 2 days. She denied fever, chills or other associated symptoms. She did not travel recently or have any sick contacts. Her past medical history was unremarkable and has had all recommended immunizations as a child. Her only medications included oral contraceptive pills. She did not smoke or abuse alcohol. Her family history was significant for myocarditis in her sister and her paternal aunt at around the same age.

The various patient parameters during the clinical course of this illness are outlined in Table 1.

**Table 1:** Various Clinical Parameters of the Case

Initial Vitals	Blood pressure: 135/80 mmHg; Heart rate: 77/min; Respiratory rate: 18/min; Temperature: 35.6 °C; O_2_ sat: 100% on room air
Initial Labs	WBC: 12.8 (74% polymorphs; 18% lymphocytes) TSH, Mg^2+^, RF, CRP, D-Dimer, ESR and Hgb were all unremarkable.
Initial Cardiac Markers	Troponin I: 109.13 ng/ml (0.00 - 0.04); Creatine Kinase: 1296 IU/L (45 - 230); CK-MB mass: 121.9 ng/ml (≤ 5); MB index: 9.4 (0.0 - 3.9)
EKG	Normal sinus rhythm with S1Q3, right axis deviation and RSr’ pattern in V1
Imaging/Nuclear studies	CT Angiogram Chest: No evidence of acute Pulmonary Embolism Ventilation/Perfusion scan: Negative for Pulmonary Embolism
Ejection Fractions during entire hospitalization and follow ups	Day 1 (at presentation): 60 - 65%; no wall motion abnormality Day 2 (day of resuscitation): 20 - 25%; no wall motion abnormality Day 3: 45%; no wall motion abnormality Day 10 (at discharge): 45%; no wall motion abnormality 2-month follow up: 50%; no other abnormalities 6-month follow up: 55%; no other abnormalities

**Figure 1:**
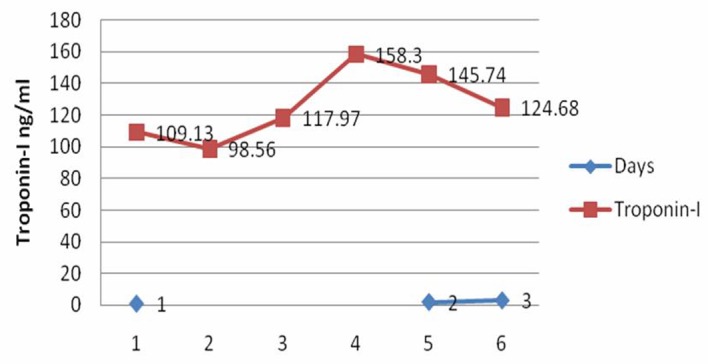
Trend of troponin-I over days of myocarditis hospitalization.

The troponin I was markedly elevated at presentation and during the first few days of hospital course (Fig. 1). Further troponin levels were not obtained during the clinical course.

**Figure 2:**
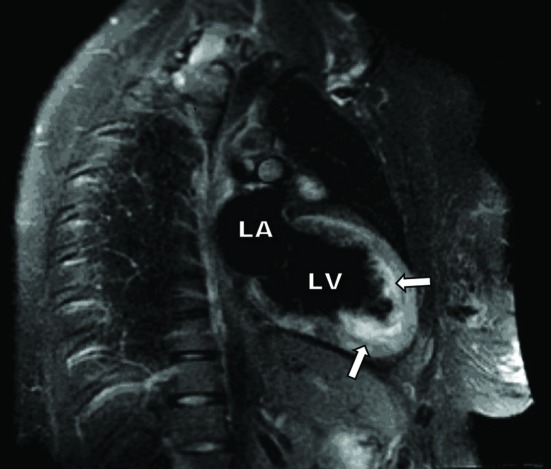
Sagital view of the heart on Cardiac MRI with T2 weighted and viability/scar imaging showing diffuse circumferential, abnormal myocardial enhancement consistent with myocarditis of the left ventricle (shown by arrows).

She had frequent premature ventricular complexes and was treated with Metoprolol. On day three she had ventricular fibrillation and suffered cardiac arrest with successful defibrillation and resuscitation. She was treated with 450 mg of intravenous Amiodarone and required mechanical ventilation. On echocardiogram, the left ventricular ejection fraction has decreased to 20 - 25% (60 - 65% at admission). The patient was transferred to tertiary care center for further management. Cardiac catheterization showed no evidence of coronary artery disease. Endomyocardial biopsy showed myocytes with no specific diagnostic abnormality and a Congo red stain was negative. MRI with T2 weighted and viability/scar imaging revealed diffuse circumferential, abnormal mid-myocardial and basilar epicardial edema and abnormal myocardial enhancement consistent with myocarditis (Fig. 2). Repeat MRI showed improving myocarditis. The left ventricular ejection fraction improved to 45% on pre-discharge echocardiogram 7 days after presentation. Patient was tapered off the Amiodarone therapy and remained stable in sinus rhythm. Patient was discharged only on Carvedilol (6.25 mg twice daily) and her course was uneventful on follow up at two and six months post discharge. The left ventricular ejection fraction has improved to 55% (at 6 months) on repeat echocardiographic evaluation.

## Discussion

Myocarditis is the inflammation of the myocardium with necrosis of the myocytes in the absence of ischemia, predominantly confined to subepicardial with less frequent involvement of midwall or transmural myocardium. Myocarditis is a manifestation of etiologies such as infections, radiation, hypersensitivity, trauma, chemical and physical agents. It can either be focal or diffuse and has varied clinical presentations with a mild chest pain lasting for a few days and resolving completely without any consequences in its mildest form, to a stormy clinical course complicated by various types of malignant arrhythmias and progression to a fulminant form characterized by acute congestive heart failure requiring management with extra corporeal membrane oxygen and cardiac transplantation to death in its severe form [1-3].

Cardiac biomarkers are elevated in about one-third of patients with acute myocarditis [4, 5] and are of help in making the clinical diagnosis [6]. Elevation of cardiac Troponin I is more common than creatine kinase MB as seen from available clinical and experimental data [5]. Troponin I has limited sensitivity (34%) in the setting of acute myocarditis [7]. The degree of elevation of the cardiac markers in acute myocarditis is directly proportional to the degree of myocardial damage, i.e., less in focal compared to diffuse form [8] and more in fulminant than in non-fulminant cases [1]. For the same reason the degree of cardiac enzyme elevation is less in the initial stages of the disease and worsens during the clinical course with greater myocardial tissue necrosis.

Marked elevation of troponin levels at presentation, especially in the absence of any evidence of left ventricular dysfunction is unusual in the clinical course of myocarditis. Our patient had troponin levels greater than 100 ng/ml at presentation with normal vital signs and left ventricular ejection fraction (Table 2).

**Table 2:** Left Ventricular Internal Diameters at Various Points During the Hospital Course

	On admission	On the day of resuscitation (day 3)	At discharge
LVIDd	4.4 cm	4.3 cm	4.3 cm
LVIDs	3.0 cm	3.9 cm	2.8 cm
LV Ejection fraction	55 - 60%	20 - 25%	45%

LVIDd: Left Ventricular Internal Diameter at the end of diastole; LVIDs: Left Ventricular Internal Diameter at the end of systole.

Initial troponin levels in myocarditis within twelve hours of presentation of only as high as ∼ 50 ng/ml was reported in various published articles [8-15] with a wide range (< 1 to 48.5 and a mean of 12.25 ng/ml). The peak troponin level even in fulminant forms of myocarditis was reported to be less than 50 ng/ml in majority of published reports [4, 9, 11, 12, 14, 16]. We found only one report describing the clinical course of four patients with fulminant myocarditis with troponin levels that peaked to ∼ 190 ng/ml in 3 survivors and ∼ 1200 ng/ml in the non-survivor. Unlike our patient, the markedly elevated peak troponin levels in these patients occurred during the clinical course rather than at presentation [1].

### Conclusion

Myocarditis with marked elevation of cardiac biomarkers at presentation and during the early initial phase of the illness prior to left ventricular dysfunction is a rare manifestation. There is limited published literature of such cases and this may represent a specific subset of myocarditis and may portend an impending complicated clinical course. It is unknown if the clinical presentation is related to etiologic agent or susceptibility due to underlying genetic variation. The management includes close hemodynamic and electrocardiographic monitoring and initiation of prophylactic low dose of beta blockers. Malignant arrhythmias and cardiogenic shock may necessitate anti-arrhythmic therapy and/or circulatory hemodynamic support therapies. Further systematic research is required to define the clinical phenotype and elucidate underlying mechanisms.

## References

[1] Oshima K, Kunimoto F, Hinohara H, Hayashi Y, Hirato J, Tajima Y, Kuwano H (2008). Fulminant myocarditis treated with percutaneous cardiopulmonary support system (PCPS). Ann Thorac Cardiovasc Surg.

[2] Lewis GD, Holmes CB, Holmvang G, Butterton JR (2007). Case records of the Massachusetts General Hospital. Case 8-2007. A 48-year-old man with chest pain followed by cardiac arrest. N Engl J Med.

[3] Graner M, Lommi J, Kupari M, Raisanen-Sokolowski A, Toivonen L (2007). Multiple forms of sustained monomorphic ventricular tachycardia as common presentation in giant-cell myocarditis. Heart.

[4] Leeper NJ, Wener LS, Dhaliwal G, Saint S, Wachter RM (2005). Clinical problem-solving. One surprise after another. N Engl J Med.

[5] Lauer B, Niederau C, Kuhl U, Schannwell M, Pauschinger M, Strauer BE, Schultheiss HP (1997). Cardiac troponin T in patients with clinically suspected myocarditis. J Am Coll Cardiol.

[6] Cooper LT (2009). Myocarditis. N Engl J Med.

[7] Smith SC, Ladenson JH, Mason JW, Jaffe AS (1997). Elevations of cardiac troponin I associated with myocarditis. Experimental and clinical correlates. Circulation.

[8] Stankewicz MA, Clements SD (2004). Fulminant myocarditis presenting with wide complex tachycardia. South Med J.

[9] Ammann P, Naegeli B, Schuiki E, Straumann E, Frielingsdorf J, Rickli H, Bertel O (2003). Long-term outcome of acute myocarditis is independent of cardiac enzyme release. Int J Cardiol.

[10] Weidenbach M, Springer T, Daehnert I, Klingel K, Doll S, Janousek J (2008). Giant cell myocarditis mimicking idiopathic fascicular ventricular tachycardia. J Heart Lung Transplant.

[11] Roongsritong C, Warraich I, Bradley C (2004). Common causes of troponin elevations in the absence of acute myocardial infarction: incidence and clinical significance. Chest.

[12] Saiki A, Iwase M, Takeichi Y, Umeda H, Ishiki R, Inagaki H, Kato Y (2007). Diversity of the elevation of serum cardiac troponin I levels in patients during their first visit to the emergency room. Circ J.

[13] Coudrey L (1998). The troponins. Arch Intern Med.

[14] Lakkireddy DR, Kondur AK, Chediak EJ, Nair CK, Khan IA (2005). Cardiac troponin I release in non-ischemic reversible myocardial injury from acute diphtheric myocarditis. Int J Cardiol.

[15] Tai YT, Lau CP, Fong PC, Li JP, Lee KL (1992). Incessant automatic ventricular tachycardia complicating acute coxsackie B myocarditis. Cardiology.

[16] Parrillo JE (2001). Inflammatory cardiomyopathy (myocarditis): which patients should be treated with anti-inflammatory therapy?. Circulation.

